# Emergency treatment of airway obstruction caused by a laryngeal neuroendocrine tumor: A case report

**DOI:** 10.1097/MD.0000000000033081

**Published:** 2023-02-22

**Authors:** Qiulei Zhang, Chengwei Zhang, Yongjie Yin

**Affiliations:** a Department of Emergency and Critical Care, The Second Hospital of Jilin University, Changchun, China; b Department of Anesthesiology, The Second Hospital of Jilin University, Changchun, China.

**Keywords:** awake intubation, case report, difficult airway, postoperative intensive care, UE visual laryngoscope

## Abstract

**Rationale:**

Laryngeal obstruction is a life-threatening adverse event that requires urgent and appropriate management, particularly in patients with coexisting cardiopulmonary and brain comorbidities. However, laryngeal obstruction caused by laryngeal neuroendocrine tumors has rarely been reported.

**Patient concerns:**

Neuroendocrine tumors can cause pathological changes in the neuro-humoral system, and asphyxia caused by airway obstruction has a more adverse effect on patients with neuroendocrine tumors.

**Diagnoses:**

We report the case of a 64-year-old man with clinical manifestations of dyspnea. Preoperative and intraoperative pathological examination indicated that the patient was diagnosed with life-threatening airway obstruction caused by a laryngeal neuroendocrine tumor, pneumonia, and scoliosis.

**Interventions:**

The patient underwent laryngeal tumor resection under general anesthesia. He was recovered well and was generally good without the necessity of undergoing radiotherapy and chemotherapy at the 6-months follow-up.

**Outcomes:**

This case report has provided an emergency treatment strategy associated with awake intubation. We concluded that flexible establishment of an artificial airway, skilled anesthesia and surgical manipulation, and necessary postoperative intensive care are extremely important for improving the prognosis of patients with severely difficult airway. It is noteworthy that the timely adjust for endotracheal intubation strategy according to the patient’s response is needed. It is important for the long-term prognosis of patients to avoid the establishment of a traumatic artificial airway and the occurrence of adverse complications.

**Lessons:**

1. Introduction; 2. Case presentation; 3. Discussion; 4. Conclusion.

## 1. Introduction

Laryngeal obstruction is a life-threatening emergency situation. In addition to laryngeal masses, factors such as age, obesity, emergency surgery, and neurological or endocrine system disorders may predict a high risk of failed and difficult intubation.^[1]^ Generally, a neuroendocrine tumor is rarely located in throat.^[2]^ However, it grows in the supraglottic region, influencing the patient’s ventilation function in this case.

## 2. Case presentation

The patient consented to the publication of this report.

The case occurred in May 2021. A 64-year-old man (height, 170 cm; body weight, 80 kg) was admitted to our hospital with a chief complaint of dyspnea for 1 month and worsened for 3 days. The patient suffered from a history of cerebral hemorrhage and hypertension for 6 years. The patient went to the local hospital and received conservative treatment without significant relief. Then he came to our hospital for further treatment. The preliminary diagnosis and therapeutic measures were clarified after a series of examinations were carried out within 2 days of admission.

**Figure 1.**
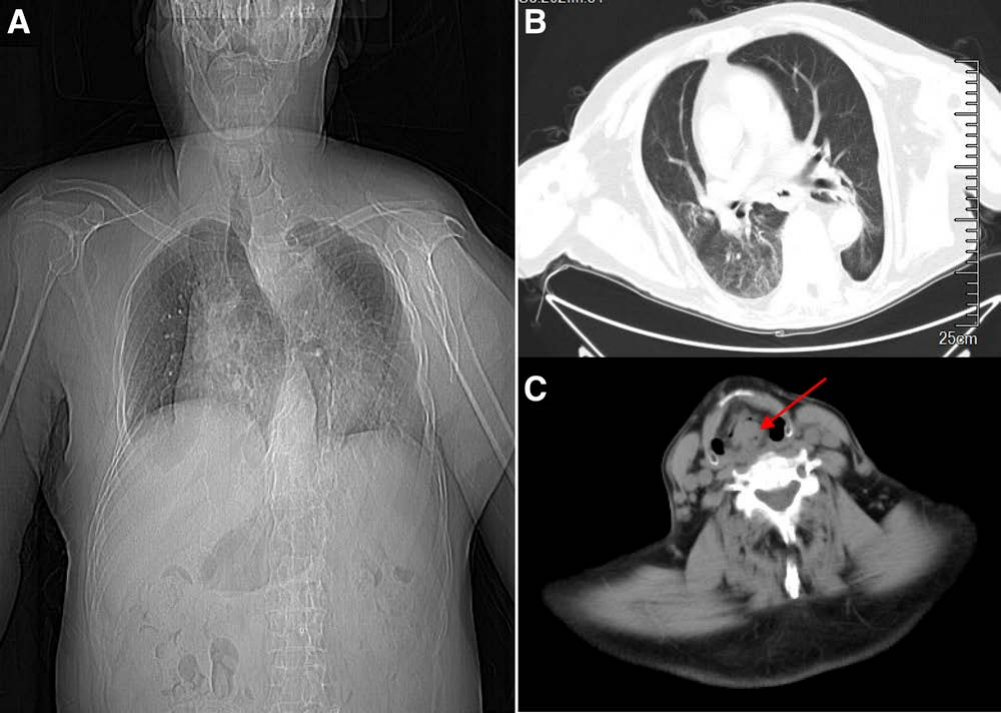
(a) The chest X-ray showed severe scoliosis. (b) The chest CT showed bilateral pneumonia. (c) Laryngeal CT showed epiglottis occupying lesion. The red arrow indicates the epiglottic neoplasm. CT = computed tomography.

Physical examination showed clear mind, hoarse voice, inspiratory laryngeal wheezing, and worsening dyspnea in supine position. Preoperative chest X-ray examination revealed severe scoliosis (Fig. 1a). Chest computed tomography displayed bilateral pneumonia (Fig. 1b). The examination of laryngeal computed tomography showed epiglottis occupying lesion and multiple lymph nodes on both sides of neck (Fig. 1c). Electronic laryngoscopy revealed the epiglottic neoplasm, which almost completely blocked the laryngeal opening. In addition, arterial blood gas analysis showed that P_a_O_2_ was 76 mm Hg and P_a_CO_2_ was 51 mm Hg. There was no obvious abnormality in electrocardiogram, echocardiography or preoperative laboratory test. The patient was diagnosed with epiglottic neoplasm, laryngeal obstruction, bilateral pneumonia and scoliosis. The nature of the neoplasm requires pathological examination to determine and emergency surgery was scheduled.

**Figure 2.**
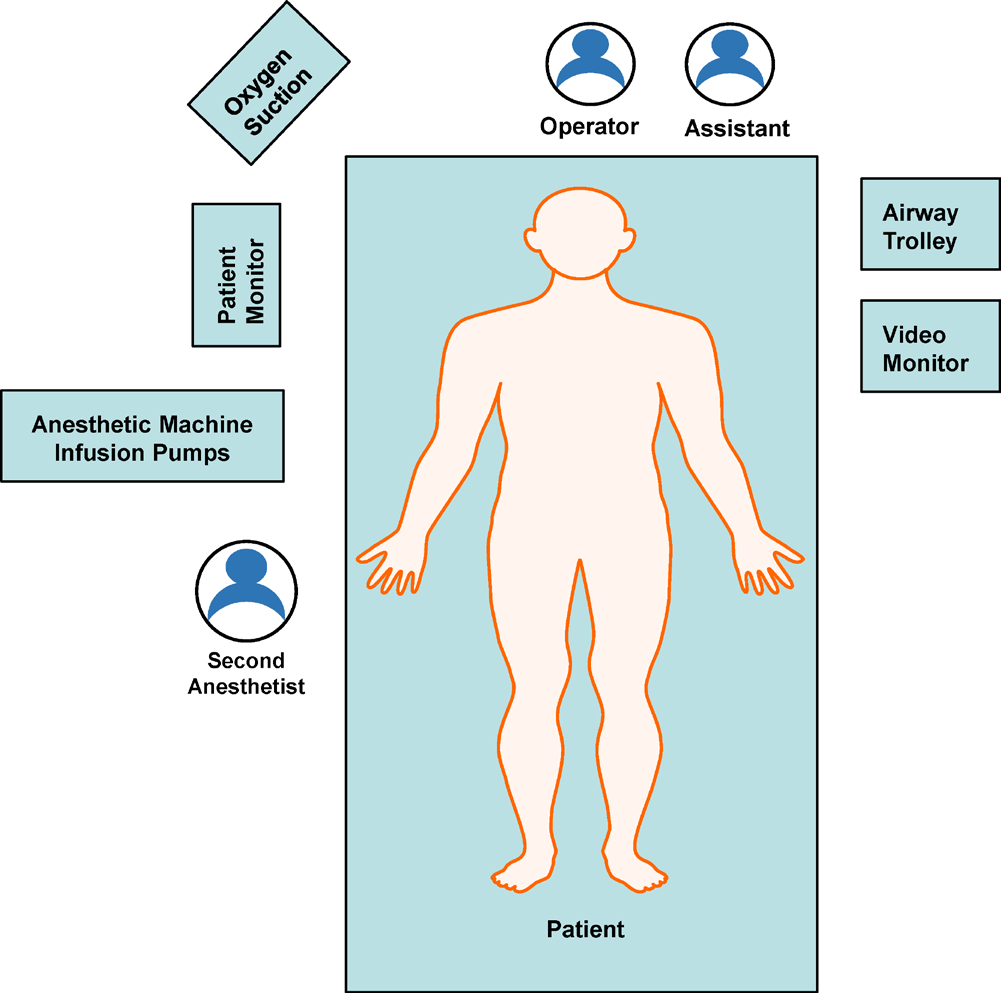
Location of personnel, equipment, and instruments during emergency surgery.

**Figure 3.**
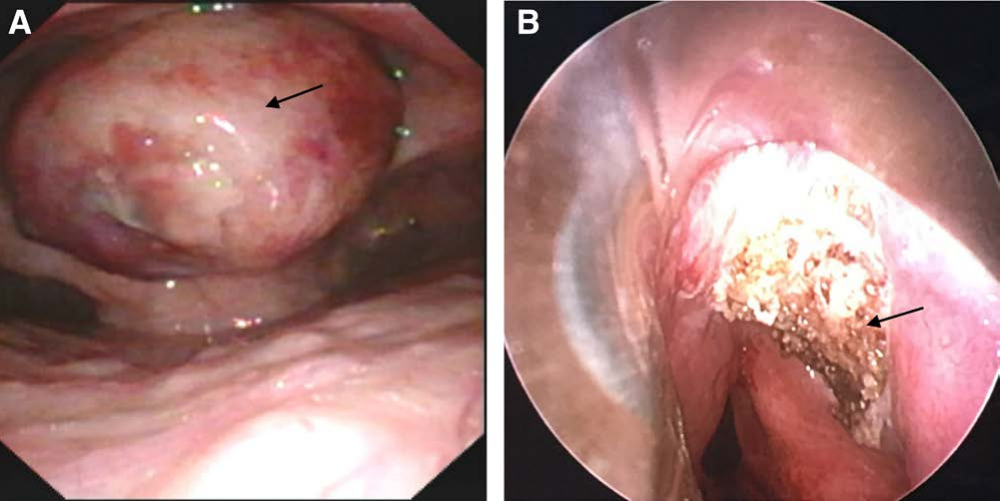
The view of the epiglottic neoplasm during emergency surgery. (a) Showed the neoplasm nearly obstructed more than 95% of the throat area. (b) Showed the neoplasm on the epiglottis was completely removed. The black arrow indicates the location of neoplasm before and after surgery.

The location of each emergency rescue personnel and the required instruments during surgery were described in the following (Fig. 2). The patient received an intravenous bolus of 0.5 μg kg^−1^ dexmedetomidine for 10 minutes after entering the operating room and then changed to a continuous infusion of 0.2 μg kg^−1^ h^−1^. The oral cavity and throat were fully topical anesthetized with 10% lidocaine aerosol. Cricothyroid membrane puncture anesthesia of the subglottal airway was performed with 2 mL 2% lidocaine. Simultaneously, 5 µg sufentanil was injected intravenously. Afterwards, a fiber bronchoscope (TIC-SD-1, UE Medical Co., Ltd., Zhejiang, China) was carefully inserted into the patient’s oropharynx under the premise of complete preoxygenation and the value of EEG bispectral index of 80, but the first attempt failed when the patient’s pulse oximetry once dropped even to 82%. Then, sufentanil (5 µg) and urapidil (5 mg) were given to achieve a better compliance of the patient and stable hemodynamics. Subsequently, the visual UE laryngoscope (TD-C-IV, UE Medical Co., Ltd.) was used to reveal glottis and showed that the huge neoplasm nearly obstructed more than 95% of the throat area. A 6.0 mm tube was selected to insert in the only visible gap with a respiratory action. Once intratracheal ventilation was confirmed by monitoring end-tidal carbon dioxide, the general anesthesia is routinely performed. Since then, the patient’s pulse oximetry has been maintained at more than 95% during the operation. Finally, the epiglottic neoplasm was completely removed by plasma radiofrequency ablation at the low temperature using a microscope (Fig. 3a and b). Intraoperative rapid pathological examination indicated the laryngeal mass as a neuroendocrine tumor. antiinfection and other symptomatic treatments are still actively given for patient with pneumonia in intensive care unit. The patient was discharged without dyspnea and was generally in good condition at 6 months postoperative follow-up without further radiotherapy and chemotherapy.

## 3. Discussion

This case report concentrated on the description of successfully awake intubation process in the emergency treatment, which reversed the patient’s dyspnea and ensured the perioperative safety. In this case, a complete effective airway management plan must be developed for such predictable difficult airway.^[3]^ The advantage of this case report is to establish an artificial airway with the aid of mild sedation, topical anesthesia, and visual intubation equipment, while maintaining spontaneous breathing. The main objective of awake sedation and topical anesthesia is to keep a state of tranquility, analgesia with a highly degree of cooperation. However, during this period, apnea is inevitable, and assisted application of continuous positive airway pressure and positive end-expiratory pressure to cope with possible hypoxia and hypercapnia is essential to avoid further arrhythmias and complete airway obstruction.

It is noteworthy that the frequency of glottis exposed by laryngoscope should be limited to 3 times, and maintenance of ventilation and oxygenation is the top priority in difficult airway management.^[4]^ Despite Cabrini et al demonstrated that a fiber bronchoscope plays an important role in difficult airway treatment, blurred vision caused by secretion and blood mainly increases the difficulty of intubation and even leads to failure.^[5]^ The endotracheal intubation was finally completed with assistance of the UE video laryngoscope. UE video laryngoscope screen visualization is conducive to magnifying the view of airway, guiding the correct placement of endotracheal intubation, and improving the success rate of intubation. To some extent, it has been shown that the video laryngoscope possesses some advantages to reveal glottis.^[6]^ While, selecting an appropriate catheter facilitates successful intubation and avoids damage to soft tissue surrounding the airway. In addition, the supraglottic airway device was found inappropriate for this patient due to the almost complete laryngeal obstruction.^[7]^

Successful endotracheal intubation avoids invasive artificial airway, which may cause infection, tracheal stenosis, and trachea-esophageal fistula, leading to lifelong pain in patients. And this patient was satisfied with the treatment received. Otherwise, other treatments, such as awakening the patient, surgery cancelation, changing to the emergency airway treatment, etc should be applied. The objective of the above-mentioned protocol is to ensure oxygenation and reduce the possibility of creating a traumatic artificial airway. At the same time, postoperative intensive care, antiinfection, and systemic oxygen therapy all contribute to perioperative safety.

## 4. Conclusion

The case of difficult airway due to a rare laryngeal neuroendocrine tumor poses a challenge to clinicians. A comprehensive preoperative evaluation suggested that the strategy of awake intubation is feasible and safe. In the future, we will attempt to improve the timeliness and effectiveness of emergency treatment, in order to benefit patients with similar difficult airways.

## Acknowledgments

We kindly thank Weinan Yuan for his assistance in the collection of image information. We also thank Tiecheng Liu, Zhimin Song and Guoliang Liu for their medical support during perioperative management.

## Author contributions

**Conceptualization:** Qiulei Zhang.

**Investigation:** Qiulei Zhang, Chengwei Zhang.

**Project administration:** Yongjie Yin.

**Resources:** Chengwei Zhang.

**Supervision:** Chengwei Zhang.

**Writing – original draft:** Qiulei Zhang.

**Writing – review & editing:** Yongjie Yin.
